# The Effect of Pre-operative Psychological Interventions on Psychological, Physiological, and Immunological Indices in Oncology Patients: A Scoping Review

**DOI:** 10.3389/fpsyg.2022.839065

**Published:** 2022-04-14

**Authors:** Tsipi Hanalis-Miller, Gabriel Nudelman, Shamgar Ben-Eliyahu, Rebecca Jacoby

**Affiliations:** ^1^School of Psychological Sciences, Tel Aviv University, Tel Aviv-Yafo, Israel; ^2^School of Behavioral Sciences, The Academic College of Tel Aviv-Yaffo, Tel Aviv-Yafo, Israel; ^3^Sagol School of Neuroscience and School of Psychological Sciences, Tel Aviv University, Tel Aviv-Yafo, Israel; ^4^Stress, Hope and Cope Laboratory, School of Behavioral Sciences, The Academic College of Tel Aviv-Yaffo, Tel Aviv-Yafo, Israel

**Keywords:** stress, surgery, pre-operative, psychological intervention, cancer, oncology

## Abstract

**Introduction:**

The stressful pre-operative period exerts a profound impact on psychological, physiological and immunological outcomes. Oncological surgeries, in particular, elicit significantly higher stress responses than most other surgeries. Managing these responses through psychological interventions may improve long-term outcomes. The purpose of the current research was to review studies that have explored pre-operative psychological interventions in cancer patients in order to map the types of current interventions and provide an initial assessment of whether these interventions improved psychological, physiological, and/or immunological indices as well as long-term cancer outcomes.

**Methods:**

A systematic literature search for studies that included pre-operative psychological interventions in oncology patients was conducted, using the databases PubMed and Web of Science. Inclusion criteria included studies pertaining to oncological surgery in adults, study designs that included a clearly defined pre-operative psychological intervention and control group.

**Results:**

We found 44 studies, each using one of the following interventions: psychoeducation, cognitive interventions, relaxation techniques, integrated approaches. All the studies reported improved immediate post-operative psychological, physiological, and/or immunological outcomes. Only a few studies addressed long-term cancer outcomes, and only one reported improved survival.

**Conclusions:**

Research on pre-operative interventions with cancer patients is missing systematic methods. Studies provide varying results, which makes it difficult to compare them and reach reliable conclusions. There is considerable heterogeneity in the literature regarding the specific intervention used, the timing of intervention, the characteristics of the patients studied and the outcome measures. In order to improve research in this field, including the measurement of long-term outcomes, we suggest some steps that should be taken in further research.

## Introduction

### Surgery as a Stressful Event

Surgery is perceived as one of the most stressful events a person confronts in their life (Gouin and Kiecolt-Glaser, 2011). While anticipating surgery, most people feel emotional distress including fears and concerns regarding: hospitalization, anesthesia and its potential complications, pain, the recovery process, potential disabilities, interactions with hospital staff and roommates, and separation from work, friends, and family members (Johnston, 1980; Gouin and Kiecolt-Glaser, 2011). Pre-operative stress responses tend to emerge approximately 6 days before surgery, escalate 2 days before surgery, and gradually decrease after the operation, partly dissipating within 5–6 days (Johnston, 1980). The return to baseline stress levels usually occurs within several weeks, depending on the type of surgery (Johnston, 1980).

While low to moderate levels of anxiety often facilitate effective coping with the expected surgery (Pritchard, 2009), high levels of anxiety may have negative consequences manifested through physiological, emotional, behavioral, or cognitive perturbations. Physiological responses may include tachycardia, hypertension, the narrowing of peripheral blood vessels, elevated body temperature, and sweating (Scott, 2004; Pritchard, 2009). Additional physiological manifestations include immunological, hematological, metabolic, and hormonal changes; the latter tend to include increased activity of the sympathetic nervous system (SNS) and the hypothalamus-pituitary-adrenal (HPA) axis as well as inflammatory responses (Desborough, 2000). High levels of pre-operative anxiety increase sensory sensitivity, which may lead to a decreased pain threshold, dizziness, and nausea (Carr et al., 2006). Pre-operative anxiety has also been associated with higher doses of intraoperative anesthetics (Maranets and Kain, 1999), higher consumption of post-operative pain medication (Caumo et al., 2002), an extended hospitalization period, a higher rate of post-operative complications (Burg et al., 2003), and chronic post-surgical pain (CPSP; Khan et al., 2016). Emotional responses to surgery include agitation, fear, depression, hopelessness, and anger, which may lead to aggressive behaviors toward the medical staff (Coussens and Werb, 2002; Antoni, 2003). Cognitive and behavioral responses include excessive levels of alertness, negative thinking, and difficulty in concentration, which may lead to difficulties or inability to follow instructions (Antoni, 2003; Pritchard, 2009).

### Oncological Surgeries

Oncological surgeries (e.g., breast, prostate and endometrial cancer surgeries) were found to elicit significantly higher pre-operative stress responses than most other surgeries (Lutgendorf et al., 2007; Garssen et al., 2010; Maggi et al., 2019), which is attributed to additional fears regarding tumor recurrence, infertility, functional modifications, body deformation, and mortality (Costanzo et al., 2007; Sciarra et al., 2018; Maggi et al., 2019; Del Giudice et al., 2020). Moreover, some studies have shown that anxiety and distress could emerge even a substantial period of time before surgery, during the biopsy phase, the diagnostic process, the decision making stage, and continue for an extended period, while undergoing various post-operative adjuvant therapies (Coussens and Werb, 2002; Antoni, 2003; Maggi et al., 2019). When discussing oncological surgeries, it is extremely important to assess the influence of surgical stress on the immune system and on long-term outcomes, manifested in tumor progression and metastasis–the main cause of death.

Ample evidence links psychological and physiological stress responses to the dysregulation of the immune system (Bartal et al., 2010; Lutgendorf and Andersen, 2015). High cortisol levels, attributed to increased pre-operative psychological stress (Bartal et al., 2010), and immune system dysfunction, were both reported among patients on the day before surgery (Lutgendorf et al., 2007; Horowitz et al., 2015). Importantly, high levels of cortisol cause immune suppression and are associated with (i) decreased production of Th1 cytokines, such as IFN-γ and IL-12 (Shaashua et al., 2017; Haldar et al., 2018), which were found to be critical regulators of NK cells activity (Antoni, 2003; Horowitz et al., 2015), and (ii) increased production of Th2 cytokines, such as IL-10. In addition, cortisol induces changes in malignant genomes, thus preventing DNA repair and promoting the survival of tumor cells (Flint et al., 2007).

### Stress-Inflammatory Responses and Tumor Progression

Having cancer, awaiting surgery, and undergoing the surgical procedure induce stress-inflammatory responses and, specifically, the release of catecholamines (CAs) and prostaglandins (PGs; Antoni and Dhabhar, 2019). CAs and PGs cause immune suppression and the promotion of additional pro-metastatic processes such as the excess release of vascular endothelial growth factor (VEGF) and IL-6 from tumor cells (Horowitz et al., 2015). Moreover, the release of CAs causes changes in the expression level of a large number of genes in both the malignant tissue and its microenvironment, which contribute to cancer growth and metastasis through various known mechanisms (Hiller et al., 2018, 2019). PGs are released by tumor cells in response to stress, CAs, and tissue damage (Antoni and Dhabhar, 2019; Jeney et al., 2019). PGs are potent immunosuppressive factors and promote tumor progression also through their direct effects on the malignant tissue and its microenvironment (Jeney et al., 2019). Overall, the release of CAs and PGs during the pre-operative and entire perioperative period has a direct effect on the malignant tissue and a synergistic immunosuppressive impact that facilitates pro-metastatic processes, as reported in both clinical studies (Flint et al., 2007; Shaashua et al., 2017; Haldar et al., 2018; Antoni and Dhabhar, 2019; Ricon et al., 2019) and translational studies (Sood et al., 2007; Garssen et al., 2010; Neeman and Ben-Eliyahu, 2013; Horowitz et al., 2015).

Interestingly, translational studies have indicated that the psychological stress that precedes surgery has an additive or even synergistic deleterious effect on the physiological trauma of surgery, leading to greater immune suppression and tumor progression (Matzner et al., 2019, 2020). Furthermore, prolonged psychological stress can lead to chronic inflammatory processes (Hanahan and Weinberg, 2011), virally induced malignant processes, and defective DNA repair–all processes that foster cancer development and metastasis (Cole et al., 2015). Accordingly, increased stress levels during different stages of cancer were reported to predict early cancer recurrence (Antoni, 2000, 2003; Antoni and Dhabhar, 2019).

It should be noted that the pre-operative period, spanning days to weeks before surgery, was found in cancer patients to induce stress-derived pro-metastatic processes that were attenuated by blocking stress-inflammatory responses (Busetto et al., 2015; Shaashua et al., 2017; Haldar et al., 2018, 2020; Hiller et al., 2019; Ricon et al., 2019).

### Interventions Aiming to Reduce Stress

In order to reduce patients’ stress levels, various stress management techniques have been employed as a way of improving psychological, physiological, and immunological indices. However, most research has focused on interventions initiated weeks, months, and 7 years after surgery and thus overlooked the critical period prior to the surgery (e.g., Spiegel et al., 2007; Andersen et al., 2008; Antoni et al., 2012). It has been shown in clinical studies that the pre-operative period presents a “window of opportunity,” during which psychological and physiological stress responses can be moderated, potentially leading to improved immune function and even to reduced risks of cancer recurrence and metastasis (Antoni, 2003; Sood et al., 2007; Garssen et al., 2010; Cole et al., 2015; Horowitz et al., 2015; Shaashua et al., 2017; Ricon et al., 2019; Haldar et al., 2020). In order to map types of current pre-operative psychological interventions with cancer patients and assess whether these interventions improved psychological, physiological, and immunological indices–a scoping review was conducted, since it both identifies and maps the available evidence on a given topic (Munn et al., 2018).

## Materials and Methods

We conducted a systematic literature search between February 2019 and August 2021, focusing on studies describing the use of pre-operative psychological interventions among oncological patients. We utilized the electronic databases PubMed and Web of Science and searched for papers in English. Keywords included the following combinations of terms: cancer; pre-operative psychological intervention; psychological preparation before oncological surgery; and presurgical cancer psychological intervention. In addition to this primary literature search, we conducted a manual search in the reference lists of the included papers and in relevant review papers (Montgomery et al., 2002; Hoon et al., 2013; Tsimopoulou et al., 2015; Waller et al., 2015; Kim et al., 2020). Search keywords and corresponding number of retrieved items can be found in Supplementary Material 1. All titles and abstracts of retrieved studies were read by the first author and were discussed with the other authors. Irrelevant studies were excluded, and relevant empirical reports and review paper were comprehensively examined.

Our inclusion criteria included: (1) a planned oncological surgery in adults (age >18 years); (2) an intervention group and appropriate control group; and (3) a defined exclusive pre-operative psychological intervention (see below for types of interventions) delivered by a healthcare provider, including pre and post-operative measures. The full PRISMA checklist for Scoping Reviews (PRISMA-ScR; Tricco et al., 2018) can be found in Supplementary Material 2.

## Results

**FIGURE 1:**
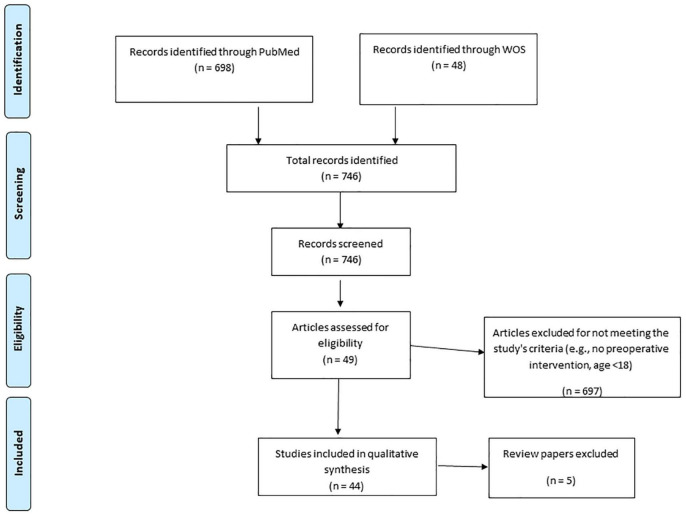
PRISMA-ScR flow diagram.

Our literature search yielded 746 papers. Of these papers, only 44 empirical reports met our inclusion criteria [see Figure 1 for a PRISMA-ScR flow diagram (Liberati et al., 2009)]. Charting form Peters et al. (2015) appears in Box 1. Other 5 review papers are discussed but were not included in the PRISMA-ScR flow diagram.

The studies reviewed reported various pre-operative psychological interventions, which we have grouped into four types based on common grouping in the field of psychology: psychoeducation (19 studies); cognitive interventions (4 studies); relaxation techniques (10 studies); and integrated approaches (11 studies). The interventions were employed at different time points and for various oncological surgeries such as breast, lung, prostate, and more. The dependent variables measured in these studies varied as well, including mainly psychological indices such as anxiety and depression; physiological indices such as post-operative complications and level of pain; and immunological indices such as cortisol levels (see Table 1). Hereby the results of the four types of interventions found in our literature review are discussed.

**TABLE 1:** Studies organized by intervention type.

Author/s (year)	Intervention type	Intervention time	Cancer type	Dependent variable	Number of participants	Number of groups	Types of groups	Time of measurement
Williams et al. (1988)	Psycho-education	-Before surgery (1–2 days)	Breast and uterus	Postoperative complications, level of self-care	60	4	Mastectomy – experimental, control group; hysterectomy –experimental, control group	Immediately after surgery, 1 month after surgery
Ali and Khalil (1989)	Psycho-education	-Before surgery (1–2 days)	Bladder	Anxiety	30	2	Experimental group, control group	3 days after surgery, before discharge (approx. 12 days)
McArdle et al. (1996)	Psycho-education	-Before surgery	Breast	General health, anxiety, depression	272	4	Routine care from ward staff, routine care plus support from breast care nurse, routine care plus support from voluntary organization, routine care plus support from nurse and organization	1 month, 3 months, 6 months, 12 months after surgery
Lilja et al. (1998)	Psycho-education	-Before surgery (1 day)	Breast	Cortisol levels, anxiety, pain	101	2	Intervention group, control group	1 day before surgery, day of surgery, 1 day after surgery, 2 days after surgery, 3 days after surgery
Katz et al. (2004)	Psycho-education	-Before surgery	Head and neck	Level of knowledge, body image, wellbeing	19	2	Psychoeducation, standard care	Baseline, pre-discharge (after surgery), 3-month follow-up
Bradley et al. (2006)	Psycho-education	-Before biopsy	Breast	Breast biopsy-related knowledge, beliefs, attitudes about breast cancer and biopsy, perceived social support	20	2	Focus group, support group	Pre-intervention, post-intervention
Allard (2007)	Psycho-education	-Before surgery (3–4 days) -After surgery (10–11 days)	Breast	Emotional distress, functional status	117	2	Experimental group, usual care (control) group	2-3 days after surgery, 9-10 days after surgery, 17-18 days after surgery
Barlési et al. (2008)	Psycho-education	-Before surgery	Lung	Quality of life	75	2	Oral only information group, oral plus written information group	Baseline, 3 months after surgery
Kakinuma et al. (2011)	Psycho-education	-Before surgery (At least 1 day)	Unknown	Anxiety, anesthesia: anesthesiologist interview time and understanding of procedure	211	2	Video group, no-video group	Before the intervention, on the day of surgery
Pinar et al. (2011)	Psycho-education	-Before surgery	Gynecologic	Anxiety	120	2	Study group – a systematic preoperative instruction, control group – routine nursing care	Before surgery, after surgery
Wysocki et al. (2012)	Psycho-education	-Before surgery (1 day)	Breast	Anxiety	58	2	Structured information (short video about practical aspects of the hospital stay, surgical and adjuvant treatment) in addition to the routine informed consent procedure for surgery, routine informed consent only	12–18 h before surgery, 24–36 h after surgery, 7 days after surgery, 30 days after surgery
Cho et al. (2013)	Psycho-education	-Before surgery (1 week) -After surgery (1 week)	Breast	Body image, comfort, knowledge, and lymphoedema	145	4	Education and Papilla Gown, education only, Papilla Gown only and control.	Baseline and 1 week and 6 months after surgery
Granziera et al. (2013)	Psycho-education	-Before anesthesia evaluation	Breast	Anxiety	234	2	Structured anesthesiology interview group (SAI), integrated multidisciplinary psycho-oncological approach (IPA)	Before randomization at baseline assessment and after anesthesiology interview
Huber et al. (2013)	Psycho-education	-Before surgery (1 day)	Prostate	Anxiety	203	2	Multimedia-supported (MME), standard education (SE)	General measurement
Angioli et al. (2014)	Psycho-education	-Before surgery (1 day)	Gynecologic	Satisfaction about preoperative received information, pain	190	2	Group V (verbal information ward) Group W (Written Information ward)	1 day after surgery, 3 days after surgery; for pain medications, every 8 h during day
O’Connor et al. (2014)	Psycho-education	-Before surgery	Rectal	Satisfaction about preoperative received information, anxiety, depression, readjustment	76	2	Intervention, control group	Baseline, after surgery, pre- discharge (Time 2), 6 months after Time 2
Lemos et al. (2019)	Psycho-education	-Before surgery (15 days)	Endometrial	Hemodynamic values and anxiety	72	2	Intervention group, control group	In Group A, the Beck anxiety inventory, blood pressure, and heart rate were evaluated before and after the preoperative education; in Group B, these parameters were evaluated at the beginning and end of the consultation
Sun et al. (2018)	Psycho-education	-Before surgery	Lung	Self-efficacy, knowledge, activation, and emotional QOL	60 (38 patients, 22 family caregivers)	2	Multimedia self-management (MSM) intervention	Before surgery (pre-intervention), on discharge, first outpatient visit after surgery (approximately 2–4 weeks post-discharge)
Pillay et al. (2020)	Psycho-education	-Before biopsy	Prostate	Anxiety, patient health, decisional conflict	98	2	Intervention group, control group	Pre-biopsy, 2–3 weeks post-biopsy
Burton et al. (1995)	Cognitive	-Before surgery (afternoon)	Breast	Anxiety, depression	295	4	(1) Preoperative interview plus a 30-minute preoperative psychotherapeutic intervention; (2) preoperative interview plus a 30-minute chat to control for the effects of attention; (3) preoperative interview only; and (4) routine hospital care control	3 months, 1 year after surgery
Oetker-Black et al. (2003)	Cognitive	-Before surgery (1–3 days)	Uterus	Anxiety, pain, health status, ambulation, vital capacity, postoperative complications, length of stay	108	2	Efficacy enhancing teaching group, usual care group	Before surgery post-intervention outcome measures, day of surgery, first day after surgery, day of discharge, 6 week follow-up, 6 month follow- up
Ganry et al. (2018)	Virtual reality (VR) – cognitive	-Before surgery	Skin	Preoperative anxiety, cortisol levels, heart coherence	10	1	Experimental hypnosis intervention	Before and after the intervention
Garcia et al. (2018)	Therapeutic listening – cognitive	-Before surgery (1 day)	Colorectal	Anxiety, surgical fears, physiological variables	50	2	Intervention group (therapeutic listening), control group	First approach, pre- and post-intervention
Rapkin et al. (1991)	Relaxation techniques	-Before surgery (1-3 days)	Head and neck	Postoperative complications, pain medication	36	2	Experimental hypnosis intervention, usual care	Before surgery, 72 h after surgery, 1–6 weeks after surgery
Enqvist et al. (1997)	Relaxation techniques	-Before surgery (4-8 days)	Breast	Pain, nausea	50	2	Control group, hypnosis group	24 h before surgery, 1–5 days after surgery, 2 weeks after surgery
Montgomery et al. (2002)	Relaxation techniques	-Before biopsy	Breast	Pain, distress	20	2	Hypnosis group, attention control group	Pre-intervention, after surgery
Haase et al. (2005)	Relaxation techniques	-Before surgery	Abdominal	Analgesic requirement, pain, pulmonary function, duration of postoperative ileus, fatigue	60	3	Guided imagery, relaxation, control	Each day for 7 days after surgery
Montgomery et al. (2007)	Relaxation techniques	-The day of surgery	Breast	Pain, nausea, fatigue	200	2	Hypnosis group, control group (standard care)	Day of the surgery (after the surgery)
Schnur et al. (2008)	Relaxation techniques	-The day of surgery	Breast	Mood states, upset, depression	90	2	15-minute pre-surgery hypnosis session, 15-minute pre-surgery attention control session	Before surgery, day of surgery
Lew et al. (2011)	Relaxation techniques	-Before surgery (1 hour)	Breast	Anxiety, distress, pain, nausea	20	1	Pre-operative Hypnosis group, historical control group	Baseline, post-intervention, after surgery
Park et al. (2013)	Relaxation techniques	-Before biopsy -The day of biopsy -The day of post-biopsy results	Breast	Stress, BSI (Brief Symptom Inventory)	40	1	Relaxation response training (RRT) group	Pre-intervention, post-intervention
Hizli et al. (2015)	Relaxation techniques	-Before surgery	Prostate	Anxiety, pain	64	2	10-min pre-surgery hypnosis session, pre-surgery control session	Before and after surgery
Zgâia et al. (2016)	Relaxation techniques	-The day of surgery	Breast	Pain	102	2	Receive relaxing technique (RT) and psychological counselling (PC); don’t receive RT and PC	Pre-intervention, during 48 h after surgery
Ambler et al. (1999)	Integrated approaches	-Before consultation with the surgeon -After consultation with the surgeon	Breast	Anxiety, depression, physical symptoms	103	2	Conventional treatment group, advocacy condition	Before surgery, 2 w after surgery, 6 weeks after surgery
Larson et al. (2000)	Integrated approaches	-Before surgery	Breast	Immune variables, health status, life orientation, depression, emotions (frequency and intensity), intrusive and avoidant thoughts and actions	41	2	Control group (standard care), intervention group	Within 1 week of diagnosis and pre-intervention, post-intervention, but within 1-3 days before surgery, 1 week after surgery
Burgio et al. (2006)	Integrated approaches	-Before surgery-Before Postoperative visit	Prostate	Decreasing the duration and severity of incontinence, and improving quality of life	125	2	Biofeedback assisted behavioral training plus daily home exercise or usual care control condition, consisting of simple postoperative instructions to interrupt the urinary stream	Before surgery, after surgery, 6 weeks, 3 months, and 6 months after surgery
Kuchler et al. (2007)	Integrated approaches	-Before surgery-A fter surgery	Gastroenterological	Quality of life, survival	271	2	Control group received standard care as provided on the surgical wards, experimental group received formal psychotherapeutic support in addition to routine care	Before surgery, 4–10 days after surgery, 3/6/12/24 months and 10 years after surgery
Parker et al. (2009)	Integrated approaches	-Before surgery (1–2 weeks)	Prostate	Psychosocial adjustment, quality of life	159	3	Stress management intervention (SM), supportive attention group (SA), standard care group (SC)	Baseline, 1 week before surgery, day of surgery, 6 weeks, 6 months, 12 months after surgery
Belleau et al. (2001)	Integrated approaches	-Before surgery (14–19 days)	Breast	Anxiety	60	2	Experimental group, control group	Before the educational intervention, immediately after the educational intervention, the day before surgery
Cohen et al. (2011)	Integrated approaches	-Before surgery (1–2 weeks)	Prostate	Mood states, immune variables	159	3	Stress management, supportive attention, standard care	General measurement
Mosher et al. (2012)	Integrated approaches	-Before surgery	Breast	Well-being, sleep disturbance, fatigue	87	2	Expressive writing, neutral writing	8-week follow-up
Garssen et al. (2010)	Integrated approaches	-Before surgery (5 days and 1 day) -After Surgery (2 days and 1 month)	Breast	Depression, anxiety, quality of life, perception of control, fatigue, pain, sleep problems, and surgery-related somatic symptoms	70	2	Intervention group, control group	Day 6 and day 1 before surgery, and day 2, 5, 30, and 90 after surgery
Zhang et al. (2013)	Integrated approaches	-Before surgery -After surgery (shortly) -Before discharge	Esophagus	Depression, anxiety, distress, survival	60	2	Intervention group (IG), control group (CG)	Before surgery, 1 week after surgery, 4 weeks after surgery, 24 weeks after surgery, 4 years after surgery
Marinelli et al. (2020)	Integrated approaches	-Before surgery (1 day)	Pancreatic	Depressive symptoms, anxiety, perceived self-efficacy, physical perceived pain, social support, fatigue, coping styles, days of hospitalization, complications	400	2	Experimental group, usual care group	Day hospital for before surgery visit (average of 1 month before surgery), day before surgery, 1 hour after the psychological intervention, after surgery (between the 3rd and 7th day after surgery)
Review manuscript:								
Hoon et al. (2013)	Psychosocial interventions	–	Colorectal	–	11 articles		–	–
Tsimopoulou et al. (2015)	Any psychological intervention	-Before surgery -After Surgery		–	7 articles	–	–	–
Montgomery et al. (2002)	Relaxation techniques	-Before surgery	–	–	20 papers, 1,624 patients	2	Control group, intervention group	–
Kim et al. (2020)	Psycho-education	-Before surgery	Meta-analysis	–	10 studies	–	–	–
Waller et al. (2015)	Psycho-education	-Before surgery		–	14 papers	2	–	–

### Psychoeducation

Anxiety levels before surgery are influenced by uncertainty regarding the anticipated medical procedures. Psychoeducation aims to provide patients with information about the surgical procedure, treatments, expected side effects, recovery process, functional modifications, etc. *via* written materials, videos, or by the medical staff. It is often assumed–but remains questionable–that patients feel less anxious when provided with such information. Our review includes 19 studies using psychoeducation interventions. Waller et al. (2015) reviewed the effects of providing information before oncological surgeries in 14 different studies. Their main conclusion was that interventions that include direct personal interaction with an information provider are the most effective: face-to-face interventions showed improvements in levels of anxiety, satisfaction and knowledge, while audio-visual and multimedia interventions advanced satisfaction and knowledge but did not reduce anxiety.

Although most studies in our review demonstrated that providing patients with information before surgery is helpful, some studies showed more complicated outcomes (Miro and Raich, 1999). A possible explanation is that individuals’ personal characteristics affect the need for information and the efficacy of preparation for surgery: specifically, “information seekers” might benefit from information before surgery, while “information avoiders” might exhibit negative effects (Miller, 1987, 1995; Prokop et al., 1991; Miro and Raich, 1999). Similarly, Shelley and Pakenham (2007) reported that a sense of self-efficacy and an external locus of control mediated the effects of pre-operative preparation among a population of cardiac patients: when external control was high, pre-operative preparation helped to reduce stress in patients with high sense of self-efficacy; when external control was low, pre-operative preparation reduced stress levels only among patients with low self-efficacy. Furthermore, it was suggested by Lilja et al. (1998) and Hoon et al. (2013) that extensive information might even have detrimental effects.

Recently, Kim et al. (2020) conducted a meta-analysis on 10 studies that showed that the effects of pre-operative education were greater in younger age groups when delivered using verbal or combined educational methods. In addition, it was found that most interventions were delivered in a single session (e.g., Lilja et al., 1998; Belleau et al., 2001; Barlési et al., 2008; Pinar et al., 2011) often 1 day prior to or on the day of surgery. This may place additional stress on patients who are already highly anxious, reducing the likelihood that the information is processed. Providing information earlier may help patients to take an active role in managing their care and enhance their preparation for the post-operative period.

Overall, these results indicate the need to provide patients with tailored information at the optimal time point in order to create a proper balance between excess and insufficient information. Delivering pre-operative education and matching expectations, can increase cancer patients’ knowledge, satisfaction, coping, and in some cases, reduce anxiety levels, especially when delivered face-to-face.

### Cognitive Interventions

Cognitive interventions before surgery focus on the early identification and processing of disruptive pre-operative beliefs and expectations in an attempt to challenge them. Our review includes four studies using cognitive interventions. These studies all deal with patients’ exercise of self-regulation cognitive skills, which were found to reduce stress, fatigue levels, pain intensity, and pain frequency (Prokop et al., 1991; Contrada et al., 2004, 2008). Likewise, identifying the concerns of women on the day before surgery and offering specific coping strategies was found to improve impaired body image (Burton et al., 1995). Oetker-Black et al. (2003) used Bandura’s self-efficacy model (Bandura, 1982) to show that an “efficacy enhancing teaching” intervention before a hysterectomy improved functioning (ambulation, vital capacity, etc.) and reduced post-operative complications including atelectasis, pneumonia, and deep vein thrombosis.

### Relaxation Techniques

Various relaxation techniques including meditation and hypnosis are used in order to reduce stress and improve coping. Our review includes 10 studies using relaxation techniques. Several studies have found that using hypnosis prior to breast biopsy or breast cancer surgery reduced the following: (1) the use of anesthetic agents during surgery and painkillers afterward; (2) post-operative side effects such as pain, nausea, vomiting, and fatigue; (3) discomfort, anxiety, depression, and emotional upset after hospital discharge; and (4) reduced use of medication (Enqvist et al., 1997; Montgomery et al., 2007; Schnur et al., 2008; Lew et al., 2011; Hizli et al., 2015; Zgâia et al., 2016). Hypnosis was also shown to have positive effects on the course of the surgery and the duration of post-operative hospitalization (Rapkin et al., 1991). However, relaxation interventions before colorectal cancer surgery were found to have no effect on post-operative clinical indices such as pain intensity or the use of analgesic drugs (Haase et al., 2005).

### Integrated Approaches

Integrated therapeutic modalities include a combination of at least two of the above categories of interventions. Our review includes 11 studies using integrated approaches. When such approaches were employed in men before prostate cancer surgery and women before breast cancer surgery, they found: (1) reduced levels of psychological distress (Ambler et al., 1999; Belleau et al., 2001); (2) improved mood states before surgery and throughout the year following surgery (Parker et al., 2009); (3) greater readiness to use mental health services if needed (Mosher et al., 2012); and (4) increased levels of circulating IL12p70 serum, natural killer cell cytotoxicity, and tumor necrosis factor (TNF)-α (Cohen et al., 2011), and decreased interferon gamma production by peripheral mononuclear cells (Larson et al., 2000).

While there are indications that pre-operative interventions contribute to short-term outcomes, there is not enough evidence regarding long-term benefits. The study of long-term cancer outcomes requires long periods of follow-up and a high number of patients to achieve sufficient statistical power (Chida et al., 2008). Attempts to study long-term cancer outcomes have only been conducted in cases where integrated pre-operative interventions were employed. Specifically, among esophageal cancer patients (*n* = 60), a treatment based on information, psychological support, stress management, and coping strategies was found to shorten duration of hospitalization, reduce the cost of medical care, and increase patients’ satisfaction with their hospital care (Zhang et al., 2013). However, 4 years after surgery, no significant differences were found between the intervention and control groups with regard to survival or health measures, as might be expected in a sample size of only 30 patients per group.

Only two studies directly examined the hypothesis that pre-operative psychological interventions might improve survival. Burton et al. (1995) provided a 30-min pre-operative personalized intervention among breast cancer patients (*n* = 295) and found reduced post-operative anxiety and distress levels. No impact on recurrence rate was found 1–2 years following surgery. In another study, Kuchler et al. (2007) implemented an individually tailored integrated intervention among 271 randomized gastroenterological cancer patients who underwent surgery between the years 1991–1993 at the University Hospital of Hamburg. Their intervention extended beyond the pre-operative timeframe and included sessions before surgery, during the entire hospitalization, and on hospital discharge. During a 10-year follow-up period, 29 of the 136 treated patients (21%) survived, compared to only 13 of the 135 (9%) control patients. This highly significant favorable effect was maintained when Cox regression analysis included all known covariates (e.g., TNM staging) (Kuchler et al., 2007). We found no other pre-operative studies that reported long-term cancer outcomes.

## Discussion

Our review exemplifies the effect of pre-operative psychological interventions among oncological patients on psychological, physiological, and Immunological Indices. However, the research in this field lacks the use of systematic methods and therefore provides varying results, which makes it difficult to compare between them and reach reliable conclusions. Moreover, there is not enough data to assess whether these interventions improve immunological measures and long-term outcomes. Although the existing research indicates that pre-operative interventions are beneficial, there is considerable heterogeneity regarding the specific intervention used, its timing, and the outcome measures (dependent variables).

### (1) Interventions

Since research designs use various interventions and it becomes difficult to compare between them, we believe that an intervention adapted protocol might contribute to decrease these limitations. Moreover, a recent research has found specific clusters of stress responses representing interpersonal variation (Jacoby et al., 2021). While planning a pre-surgical intervention, it is worth considering each person’s stress response profile and offering them a tailored intervention.

### (2) Time Points

Since there is significant variability regarding time points of interventions, measurements, and follow-up, we wish to emphasize the benefits of developing a protocol which might reduce the variability among these variables.

### (3) Dependent Variables

The multiplicity of dependent variables in the studies reviewed does not allow for clear conclusions. A focus on common variables will enable both a comparison and a synthesis of studies’ results. Recent developments in psychoneuroimmunology enable the use of research methods and indices that were not previously available (Slavich, 2020).

### (4) Samples

The sample sizes in the studies reviewed were mostly small (10–203, median 71). Six studies had higher participant number (211–400). Therefore, in most studies the statistical power for testing hypotheses regarding long-term cancer outcomes is inadequate. Studies assessing such outcomes may necessitate hundreds of patients per group according to cancer type.

## Conclusion

Scoping reviews are designed to provide an overview of the existing evidence base, regardless of quality (Peters et al., 2015). Our review shows that although there are some indications that pre-operative psychological interventions among oncological patients may improve psychological and physiological indices post-operatively, major steps should be taken to improve research in this field. We hope that our findings stimulate specific research questions including the synthesis of effect sizes and incorporation of risk-of-bias assessments. It is possible that lack of sufficient awareness of the importance of the short pre-operative period in determining long-term cancer outcomes has reduced the incentive to intervene at this stage, thus resulting in relatively few reported studies. In addition, interventions during the pre-operative period may be inconvenient to the patient and the medical establishment, thus contributing to the reduced incentive to intervene and conduct research at this point. Nonetheless, the National Comprehensive Cancer Network [NCCN] (2018) has recently acknowledged the importance of psychological interventions before surgery. Their implementation will, we believe, enhance patients’ physiological and psychological health.

The Covid 19 pandemic during 2020–2022 has raised stress levels among most populations, but especially in people who cope with bodily illness and/or are dependent on the medical services. Unfortunately, some populations, such as the elderly and lower-class people, who have limited excess to health services and cannot afford private medicine, are exposed to greater risk and suffering (Chung et al., 2015). Additionally, perioperative routine medical treatments may need reconsideration to accommodate to needed medical procedures and to prevent various risks including disease transaction (Salciccia et al., 2020a,b, 2021). Therefore, future perioperative interventions should take into consideration the impact of such periods on psychological, physiological, and immunological variables.

## Limitations

Only psychological interventions alone may lack the capacity to mitigate the stress and inflammatory responses that originate from physiological elements in the perioperative period, such as tissue damage and other physiological perturbations. Therefore, additional interventions, such as medications, should be considered in order to estimate the influence of a combination of psychological and medical interventions on long-term outcomes. Notably, recent biomarker pharmacological studies have indicated a reduction in pro-metastatic malignant potential *via* pre-operative anti-stress drug interventions. Pharmacological treatments during surgery (Shaashua et al., 2017; Haldar et al., 2018, 2020; Hiller et al., 2019; Knight et al., 2020) may be needed to overcome the impact of tissue damage and other intraoperative procedures that may mask the beneficial effects of stress-reducing psychological interventions. The separate and combined effectiveness of psychological and pharmacological approaches should thus be studied perioperatively.

Moreover, our review focuses on the pre-operative period. However, there are indications to include the interventions employed during the whole perioperative period (days before and after surgery). Several translational and clinical studies have suggested that the pre-operative period, the surgical procedure, and the immediate post-operative period each have a distinct impact on long-term cancer outcomes. For example, animal studies have clearly indicated the cumulative effects of stress and surgery (including anesthesia, tissue damage, pain, etc.) on cancer progression (Cole et al., 2015; Horowitz et al., 2015; Ricon et al., 2019). Recent clinical studies have provided similar evidences that are based on interim biomarkers and correlative evidence (Shaashua et al., 2017; Haldar et al., 2018, 2020; Hiller et al., 2019; Ricon et al., 2019; Knight et al., 2020). Consequently, it becomes clear that the perioperative period as a whole entails heightened risks for cancer progression but also offers unexploited treatment targets for improving resistance to cancer metastasis (Hiller et al., 2018; Matzner et al., 2019; Ben-Eliyahu, 2003; Benish et al., 2008)–the leading cause of cancer mortality (Mehlen and Puisieux, 2006). We therefore suggest that further research on humans should focus on the impact of interventions conducted during the entire perioperative time frame.

## Data Availability Statement

The original contributions presented in the study are included in the article/Supplementary Material, further inquiries can be directed to the corresponding author.

## Author Contributions

TH-M: conceptualization, project administration, investigation, writing – original draft, methodology, and writing – review and editing. GN: formal analysis, methodology, investigation, and writing – review and editing. RJ: conceptualization, project administration, investigation, writing – original draft, writing – review and editing, and supervision. SB-E: investigation, writing – original draft, methodology, and writing – review and editing. All authors contributed to the article and approved the submitted version.

## Conflict of Interest

The authors declare that the research was conducted in the absence of any commercial or financial relationships that could be construed as a potential conflict of interest.

## Publisher’s Note

All claims expressed in this article are solely those of the authors and do not necessarily represent those of their affiliated organizations, or those of the publisher, the editors and the reviewers. Any product that may be evaluated in this article, or claim that may be made by its manufacturer, is not guaranteed or endorsed by the publisher.
