# Supplementary material for: The Effect of Pre-operative Psychological Interventions on Psychological, Physiological, and Immunological Indices in Oncology Patients: A Scoping Review
Source: Front Psychol. 2022 Apr 14;13:839065. doi: 10.3389/fpsyg.2022.839065 (PMC9094613; doi:10.3389/fpsyg.2022.839065)
Supplement: Supplementary file 1 [file Table_1.pdf]

**Electronic databases**	**Keywords**	**No. of papers**
Web of Science	cancer; preoperative psychological intervention	18
	psychological preparation before oncological surgery	1
	presurgical cancer psychological intervention	30
PubMed	cancer; preoperative psychological intervention	399
	psychological preparation before oncological surgery	12
	presurgical cancer psychological intervention	299
